# FOXC1 induces cancer stem cell-like properties through upregulation of beta-catenin in NSCLC

**DOI:** 10.1186/s13046-018-0894-0

**Published:** 2018-09-06

**Authors:** Sisi Cao, Zhuo Wang, Xiujuan Gao, Wenjuan He, Yue Cai, Hui Chen, Rong Xu

**Affiliations:** 1 Department of Pharmacology, School of Basic Medicine, Tongji Medical College, Huazhong University of Science and Technology, Wuhan, 430030 Hubei China; 2 The Key Laboratory for Drug Target Researches and Pharmacodynamic Evaluation of Hubei Province, Wuhan, 430030 Hubei China

**Keywords:** FOXC1, Cancer stem cell-like properties, Beta-catenin, NSCLC

## Abstract

**Background:**

Accumulating evidence suggests that cancer stem cells (CSCs) play a critical role in tumor initiation, progression and therapy, and recent studies have indicated that Forkhead box C1 (FOXC1) is strongly associated with CSCs. This study investigates the regulatory effects of FOXC1 on CSC-like properties in non-small cell lung cancer (NSCLC).

**Methods:**

We analyzed FOXC1 expression in NSCLC using the Cancer Genome Atlas (TCGA) database on UALCANC and performed survival analyses of NSCLC patients on Human Protein Atlas. CSC-like properties were analyzed based on CSC marker-positive cell population, self-renewal ability, stemness-related gene expression, tumorigenicity and drug resistance. The percentage of CD133^+^ cells was analyzed by flow cytometric analysis. Self-renewal ability was detected by sphere-formation analysis. Real-time PCR, western blotting and immunohistochemical staining were employed to detect mRNA and protein levels. Tumorigenicity was determined based on a xenograft formation assay, and effects of FOXC1 on drug resistance were assessed by cell viability and apoptosis assays. Luciferase reporter and chromatin immunoprecipitation (ChIP) assays were used to investigate the binding of FOXC1 to beta-catenin promoter.

**Results:**

FOXC1 expression was found to be elevated in NSCLC tissues and negatively correlated with patient survival. FOXC1 knockdown reduced CD133^+^ cell percentage, suppressed self-renewal ability, decreased expression of stemness-related genes (Oct4, NANOG, SOX2 and ABCG2) and inhibited NSCLC cell tumorigenicity in vivo. Moreover, FOXC1 knockdown increased cisplatin and docetaxel sensitivity and reduced gefitinib resistance, whereas FOXC1 overexpression enhanced CSC-like properties. Luciferase reporter and ChIP assays showed beta-catenin to be a direct transcriptional target of FOXC1. Furthermore, overexpression of beta-catenin reversed the CSC-like property inhibition induced by FOXC1 knockdown, and knockdown of beta-catenin attenuated the CSC-like properties induced by FOXC1 overexpression.

**Conclusions:**

This study demonstrates that FOXC1 induces CSC-like properties in NSCLC by promoting beta-catenin expression. The findings indicate that FOXC1 is a potential molecular target for anti-CSC-based therapies in NSCLC.

## Background

Lung cancer is the most common cancer and is the leading cause of cancer death [1]. Despite rapid advances in cancer treatment, the prognosis of lung cancer patients remains less than optimal. The 5-year survival rate in lung cancer patients is 4–17%, depending on tumor stage and region [2, 3]. Cancer stem cells (CSCs), a small population of cancer cells that retain stem cell properties [4, 5], play an essential role in tumor initiation, progression and therapy. The self-renewal and differentiation of CSCs are responsible for tumor initiation and maintenance. CSCs also act as tumorigenic cells in metastasis. Moreover, CSCs exhibit resistance to anticancer drugs, and CSCs remaining after therapy lead to cancer recurrence. Because of the conspicuous role of CSCs in tumor initiation, maintenance, metastasis, drug resistance and recurrence, inhibiting CSCs by targeting signaling pathways that regulate these cells is an effective anti-lung cancer strategy [6].

Forkhead box (FOX) proteins, characterized by a winged helix DNA-binding domain [7], are important transcription factors [8, 9], and many FOX family members play a critical role in cancer progression [10–13]. Previous studies have proven that FOX proteins are strongly associated with CSCs. For example, downregulation of FOXA1 expression promotes CSC-like properties in breast cancer cells, leading to tamoxifen resistance [14]. FOXM1 increases the CSC population and induces endocrine resistance in breast cancer [15], and FOXQ1 inhibition with diallyl disulfide is a novel strategy for suppressing breast CSCs [16]. Forkhead box C1 (FOXC1), a member of the FOX protein family, acts as an oncogene in various cancers [17]. For instance, FOXC1 mRNA and protein levels are upregulated in 60% and 63.3%, respectively, of patients with non-small cell lung cancer (NSCLC), which is the main type of lung cancer, and FOXC1 knockdown inhibits proliferation and metastasis in NSCLC [18–20]. FOXC1 also promotes proliferation, migration, invasion and drug resistance in hepatocellular carcinoma [21, 22] and breast cancer [23–26]. Recently, several studies have shown that FOXC1 governs hair follicle stem cell quiescence to maintain regenerating potential [27, 28]. Hedgehog and Notch are essential pathways in CSC regulation [29]. FOXC1 activates Notch pathway by directly regulating Dll4 in endothelial cells [30] and enhances Hedgehog signaling activity via Gli2 binding in basal-like breast cancer [31]. Moreover, FOXC2 has been shown to enhance CSC-like properties in breast cancer and prostate cancer [32, 33]. These findings emphasize the role of FOX proteins in regulating CSCs.

Nonetheless, there is no report on the function of FOXC1 in the regulation of CSCs in NSCLC. This study is the first to investigate FOXC1 induction of CSC-like properties in NSCLC, and we hope that the findings will provide a foundation for NSCLC therapy via CSC eradication.

## Methods

### Cells and cell culture

HBE, A549, NCI-H292, NCI-H1299, NCI-H1975 and HCC827 cells were purchased from the Cell Bank of Type Culture Collection of the Chinese Academy of Sciences (China). PC9 cells were purchased from the RIKEN BioResource Center (Japan). The gefitinib-resistant PC9/G cell line was produced in our laboratory following a previously described procedure [34]. All cell lines were cultured as described in the providers’ instructions and were authenticated via short tandem repeat (STR) genotyping analysis.

### Real-time PCR

Total RNA was extracted from NSCLC cells using RNApure (ZOMANBIO, China) and reverse-transcribed into cDNA using a Reverse Transcriptase Kit (ZOMANBIO). HSYBR qPCR Mix (ZOMANBIO) was used to perform real-time PCR with primers for FOXC1 (HQP005629), beta-catenin (HQP003539), NANOG (HQP019390), Oct4 (HQP464025), SOX2 (HQP017628), ABCG2 (HQP022745) and GAPDH (HQP064347) purchased from GeneCopoeia (USA). The primers used for putative FOXC1 binding site 2 were as follows: forward, 5′-AAAAAATTGGAGGCTGCTT-3′; reverse, 5′-CCAAAGAAAAATCCCCACA-3′. Fold change was calculated by the ΔΔCt method.

### Western blotting

RIPA (Beyotime, China) buffer supplemented with phosphatase inhibitor (Beyotime) and a Nuclear Protein Extraction Kit (Beyotime) were used to extract total and nuclear proteins from NSCLC cells. The extracted proteins were transferred to PVDF membranes after fractionation by SDS-PAGE. After blocking in 5% milk for 2 h, the PVDF membranes were incubated at 4 °C overnight with a specific antibody. Protein bands were visualized using ExPlus ECL (ZOMANBIO) with a MicroChemi system (DNR, Israel). The primary antibody against glycogen synthase kinase 3 beta (GSK3β) (12456 T) was purchased from Cell Signaling Technology (USA), and primary antibodies against FOXC1 (ab5079), LMNB1 (ab133741), ABCG2 (ab207732), SOX2 (ab92494), Oct4 (ab181557), phospho-GSK3β (pGSK3β) (ab75814) and NANOG (ab109250) were purchased from Abcam (USA). The primary antibody against GAPDH (60004–1-Ig) was purchased from Proteintech (China), and the primary antibody against beta-actin (A01011–1) was purchased from Abbkine (China).

### Establishment of stable cell lines

A short hairpin RNA against FOXC1 (shFOXC1)-expressing plasmid HSH005629–33-LVRU6rLP (GeneCopoeia) or FOXC1-expressing plasmid CS-X0042-Lv217–01 (GeneCopoeia) was packaged into lentivirus following the user manual of the Lenti-Pac HIV Expression Packaging kit (GeneCopoeia). NSCLC cells were infected with lentiviral particles for 12 h. FOXC1-knockdown (A549-LV-shFOXC1 and PC9/G-LV-shFOXC1) and overexpressing (NCI-H1299-LV-FOXC1 and PC9-LV-FOXC1) cell lines were obtained by selection with puromycin. Beta-catenin-overexpressing (A549-shFOXC1-LV-beta-catenin and PC9/G-shFOXC1-LV-beta-catenin) and knockdown (NCI-H1299-FOXC1-LV-shbeta-catenin and PC9-FOXC1-LV-shbeta-catenin) cell lines were established using a similar procedure. The beta-catenin-expressing plasmid EX-T0573-Lv220 and the shbeta-catenin-expressing plasmid HSH054811–4-LVRU6GP were purchased from GeneCopoeia. The shFOXC1 target sequence was GGGAAACTGTATTAATCTTAT and the shbeta-catenin target sequence was GCTGATATTGATGGACAGTAT.

### Flow cytometric analysis

A sample of 1 × 10^6^ cells was washed twice with PBS supplemented with 0.5% BSA and 2 mM EDTA and incubated in diluted CD133-PE antibody solution (Miltenyi Biotec, Germany) for 10 min. After washing the cells twice, the percentage of CD133^+^ cells was analyzed by flow cytometry (BD, USA).

### Sphere-formation analysis

A total of 5 × 10^3^ cells were seeded into 6-well ultra low-attachment plates and incubated in DMEM/F12 (Gibco, USA) supplemented with EGF (20 ng/mL, Peprotech, USA), FGF-basic (20 ng/mL, Gibco) and B27 (20 μl/mL, Gibco) for two weeks. The number of spheres (diameter > 100 μm) was counted under an electron microscope (Nikon, Japan).

### Xenograft assay

BALB/c nude mice, aged 5 weeks and weighing 20–22 g, were purchased from Hunan SJA Laboratory Animal Co., Ltd. (China). A series of NSCLC cells (5 × 10^5^, 5 × 10^4^ and 5 × 10^3^) were suspended in Matrigel (BD, USA) and subcutaneously inoculated into the mice. The tumor volume was calculated using the formula (length × width^2^)/2.

### Immunohistochemical staining

FOXC1 and beta-catenin protein levels in xenograft tumors were detected using anti-FOXC1 (ab5079) and anti-beta-catenin (Sc-7199) antibodies following standard protocols for immunohistochemical staining.

### Cell viability assay

Cells (5 × 10^3^) were incubated in culture medium supplemented with serial concentrations of cisplatin, docetaxel or gefitinib for 48 h. A cell counting kit (ZOMANBIO) was used to measure cell viability.

### Apoptosis assay

A total of 1.5 × 10^5^ cells were incubated in culture medium supplemented with cisplatin, docetaxel or gefitinib for 48 h. The percentage of apoptotic cells was analyzed using an Annexin V-FITC apoptosis analysis kit (Sungene Biotech, China) following the user manual.

### Luciferase reporter assay

The full-length FOXC1 cDNA was cloned into the pIRES2 vector (Clontech, USA) to construct FOXC1-expressing plasmid pIRES2-FOXC1. The wild-type or mutant beta-catenin promoter [(− 1369/+ 163) beta-catenin, relative to the transcriptional start site] was cloned into the pGL3 plasmid (Promega, USA) to construct beta-catenin reporter plasmid pGL3-beta-catenin. 293 T cells were transfected with pIRES2-FOXC1, pGL3-beta-catenin and Renilla luciferase reporter PRL-TK plasmids using Lipofectamine 3000 (Invitrogen). After 48 h, a dual-luciferase reporter assay system (Promega) was employed to measure luciferase activity.

### Chromatin immunoprecipitation (ChIP)

Formaldehyde (1%) was used to crosslink cells (4 × 10^6^) at 4 °C for 12 min. Glycine (0.125 mol/L) was added to stop the crosslinking, and chromatin was sheared into small fragments using sonication. The anti-FOXC1 antibody (ab5079) and protein G beads were applied to pull down the target protein, and proteinase K was used to digest proteins at 45 °C for 50 min. Target protein-bound DNA was harvested and purified using HSYBR qPCR Mix (ZOMANBIO).

### Statistical analysis

Quantitative results are presented as the mean ± standard deviation (SD). Student’s t-test and the χ^2^ test were utilized to determine statistical significance. The Kaplan-Meier method and log-rank test were used in survival analyses. *p* < 0.05 was considered statistically significant.

## Results

### FOXC1 expression is elevated in NSCLC tissues and negatively correlates with survival probability

**Fig. 1:**
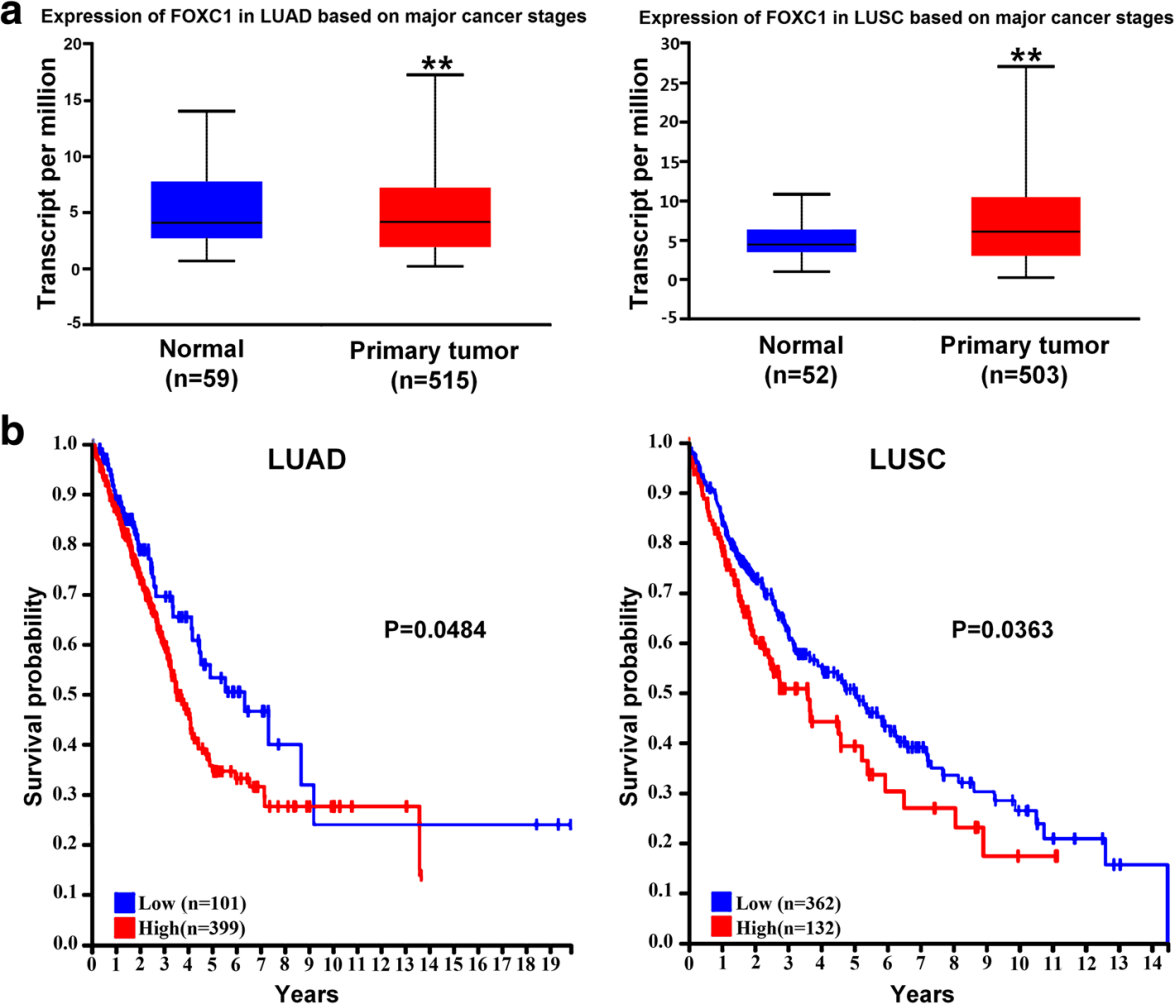
FOXC1 expression is elevated in NSCLC tissues and negatively correlates with survival probability. **a** FOXC1 expression levels in LUAD and LUSC tissues and normal lung tissues in TCGA were analyzed on UALCANC. **b** Kaplan-Meier analyses of survival probabilities of LUAD and LUSC patients were performed on Human Protein Atlas. The log-rank test was used to calculate *p* values. **P < 0.01

We analyzed FOXC1 expression in NSCLC based on information in the Cancer Genome Atlas (TCGA) database on UALCANC [35] and found elevated FOXC1 expression in lung adenocarcinoma (LUAD) and lung squamous cell carcinoma (LUSC) compared to normal lung tissues (Fig. 1a). We also examined the relationship between FOXC1 expression and NSCLC patient survival on Human Protein Atlas [36], which revealed an inverse correlation between FOXC1 expression and survival in LUAD and LUSC patients (Fig. 1b).

### FOXC1 enhances stemness of NSCLC cells in vitro

**Fig. 2:**
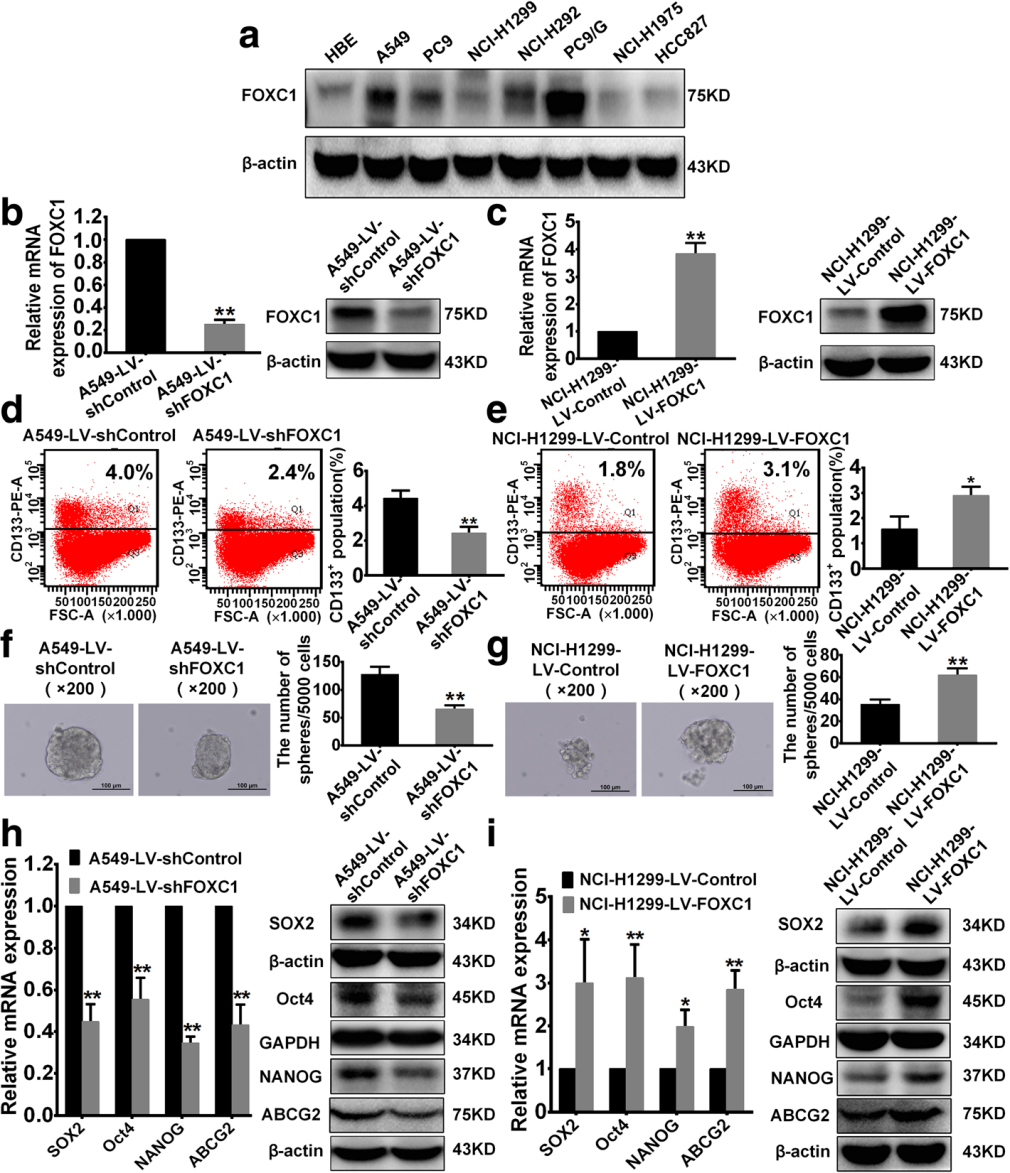
FOXC1 induces stemness of NSCLC cells in vitro. **a** FOXC1 protein levels in NSCLC cells were detected by western blotting. **b** and **c** FOXC1 mRNA and protein levels were stably downregulated in A549 cells and upregulated in NCI-H1299 cells. **d** and **e** The percentage of CD133^+^ cells was analyzed by flow cytometry. **f** and **g** Representative images (left) and numbers (right) of spheres (diameter > 100 μm). **h** and **i** Protein and mRNA levels of SOX2, Oct4, NANOG and ABCG2. All experiments were independently repeated three times. The bar graph presents the mean ± SD. *P < 0.05, **P < 0.01

We found FOXC1 to be widely expressed in NSCLC cells, and FOXC1 expression was significantly higher in gefitinib-resistant PC9/G cells than in gefitinib-sensitive PC9 cells (Fig. 2a). High (A549 and PC9/G) and low (NCI-H1299 and PC9) FOXC1-expressing cell lines were used for further studies. We established an A549-LV-shFOXC1 stable cell line with stable knockdown of FOXC1 expression (Fig. 2b), and a NCI-H1299-LV-FOXC1 stable cell line with constant FOXC1 expression (Fig. 2c). FOXC1 knockdown reduced the percentage of CD133^+^ cells (Fig. 2d), inhibited sphere formation (Fig. 2f) and downregulated mRNA and protein levels of stemness-related genes (SOX2, Oct4, NANOG and ABCG2) (Fig. 2h). Conversely, FOXC1 overexpression increased the CD133^+^ cell percentage (Fig. 2e), promoted sphere formation (Fig. 2g) and upregulated mRNA and protein levels of SOX2, Oct4, NANOG and ABCG2 (Fig. 2i).

### FOXC1 enhances tumorigenicity of NSCLC cells in vivo

**Fig. 3:**
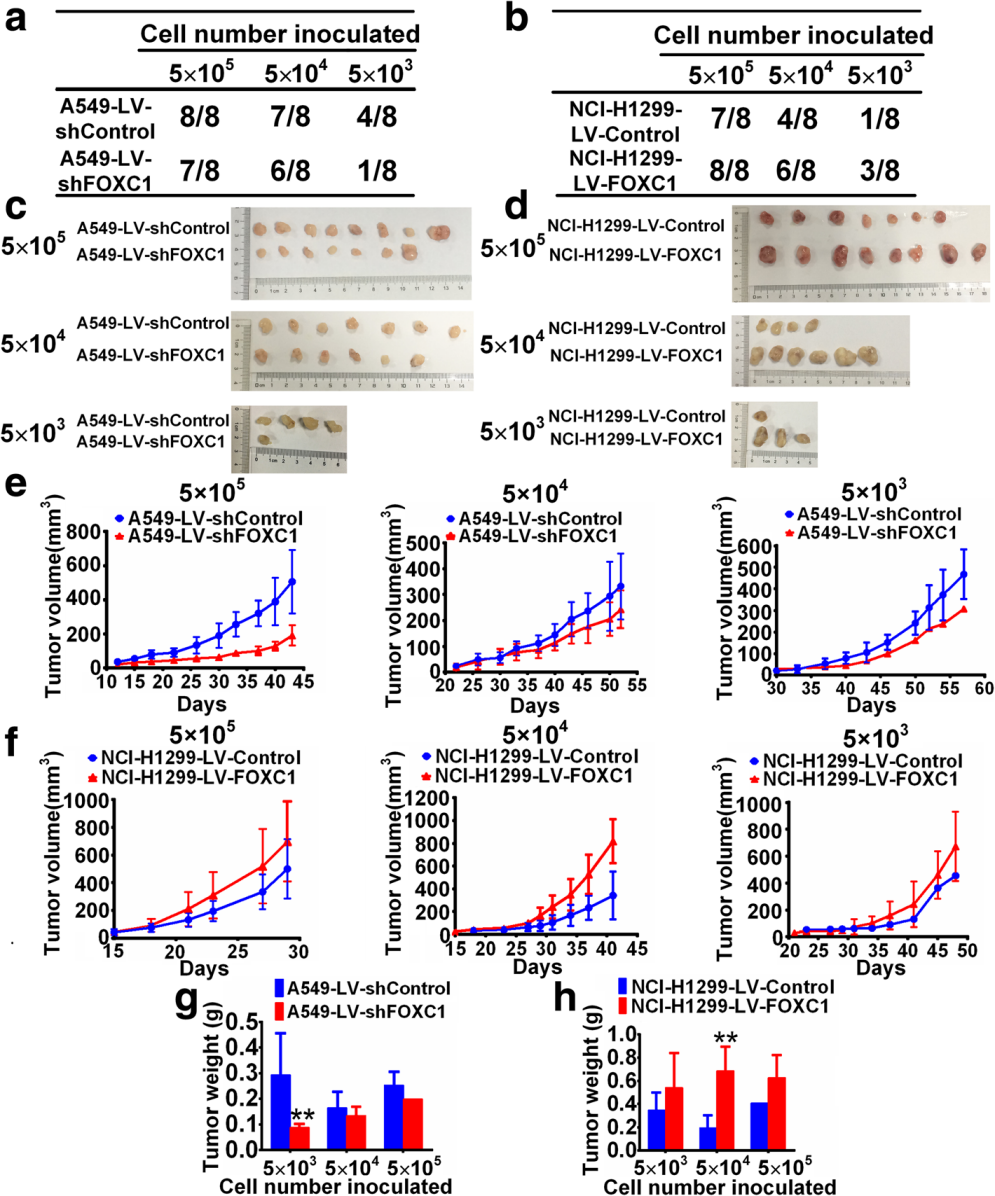
FOXC1 enhances the tumorigenicity of NSCLC cells in vivo. A series of cells (5 × 10^5^, 5 × 10^4^ and 5 × 10^3^) were subcutaneously inoculated into BALB/c nude mice (*n* = 8/group). **a** and **b** The tumor incidence of each group. **c-f** Images and growth curves of tumor xenografts. **g** and **h** Histograms show the tumor weights of each group. The bar graph presents the mean ± SD. **P < 0.01

To investigate whether FOXC1 influences NSCLC cell tumorigenicity in vivo, we subcutaneously inoculated a series of NSCLC cells (5 × 10^5^, 5 × 10^4^ and 5 × 10^3^) into BALB/c nude mice. FOXC1 knockdown decreased tumor incidence rate (Fig. 3a), tumor volume (Fig. 3c and e) and tumor weight (Fig. 3g), whereas, FOXC1 overexpression had the opposite effects (Fig. 3b, d, f and h).

### FOXC1 confers drug resistance in NSCLC cells

**Fig. 4:**
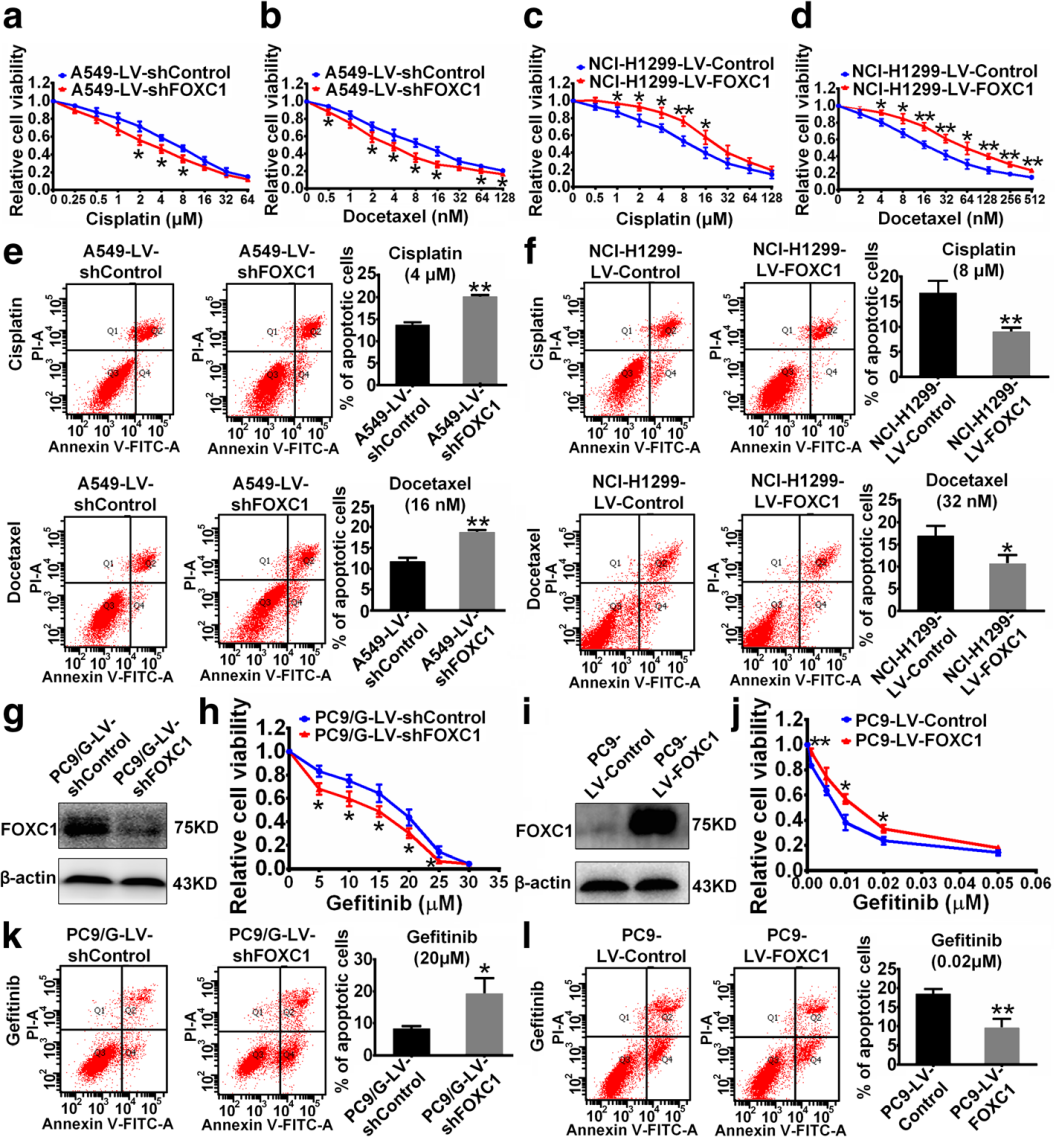
FOXC1 induces resistance to cisplatin, docetaxel and gefitinib. **a-d** Cell viability was detected after cells were treated with cisplatin or docetaxel at the indicated concentrations. **e** and **f** Percentage of apoptotic cells, including early apoptotic cells (Q2) and late apoptotic cells (Q4), was determined by flow cytometric analysis after treatment with cisplatin or docetaxel. **g** and **i** FOXC1 protein levels were stably downregulated in PC9/G cells and upregulated in PC9 cells. **h** and **j** Cell viability was detected after cells were treated with gefitinib at the indicated concentrations. **k** and **l** Percentage of apoptotic cells was determined after treatment with gefitinib. All experiments were independently repeated three times. The bar graph presents the mean ± SD. *P < 0.05, **P < 0.01

As the presence of CSCs is one of the major causes of resistance to therapy [37], we investigated whether FOXC1 is involved in drug resistance in NSCLC. Cisplatin and docetaxel are widely used cytotoxic anti-cancer agents in NSCLC treatment [38, 39]. FOXC1 knockdown enhanced the cell killing effects of cisplatin and docetaxel on A549 cells (Fig. 4a and b) and increased the percentage of apoptotic cells (Fig. 4e). In contrast, FOXC1 overexpression attenuated cisplatin and docetaxel-mediated killing of NCI-H1299 cells (Fig. 4c and d) and reduced apoptotic cell percentage (Fig. 4f). Gefitinib is a classic molecularly targeted anti-NSCLC agent [40] and FOXC1 expression was significantly higher in the gefitinib-resistant PC9/G cell line than in the gefitinib-sensitive parental PC9 cell line. We established a PC9/G-LV-shFOXC1 stable cell line, in which FOXC1 expression was stably downregulated in PC9/G cells (Fig. 4g), and a PC9-LV-FOXC1 stable cell line, in which FOXC1 expression was stably upregulated in PC9 cells (Fig. 4i). FOXC1 knockdown enhanced PC9/G cell killing by gefitinib (Fig. 4h) and increased the percentage of gefitinib-induced apoptotic cells (Fig. 4k), whereas FOXC1 overexpression attenuated the inhibitory effect of gefitinib on PC9 cell viability (Fig. 4j) and reduced the percentage of gefitinib-induced apoptotic cells (Fig. 4l).

### Beta-catenin mediates FOXC1-induced CSC-like properties in NSCLC

**Fig. 5:**
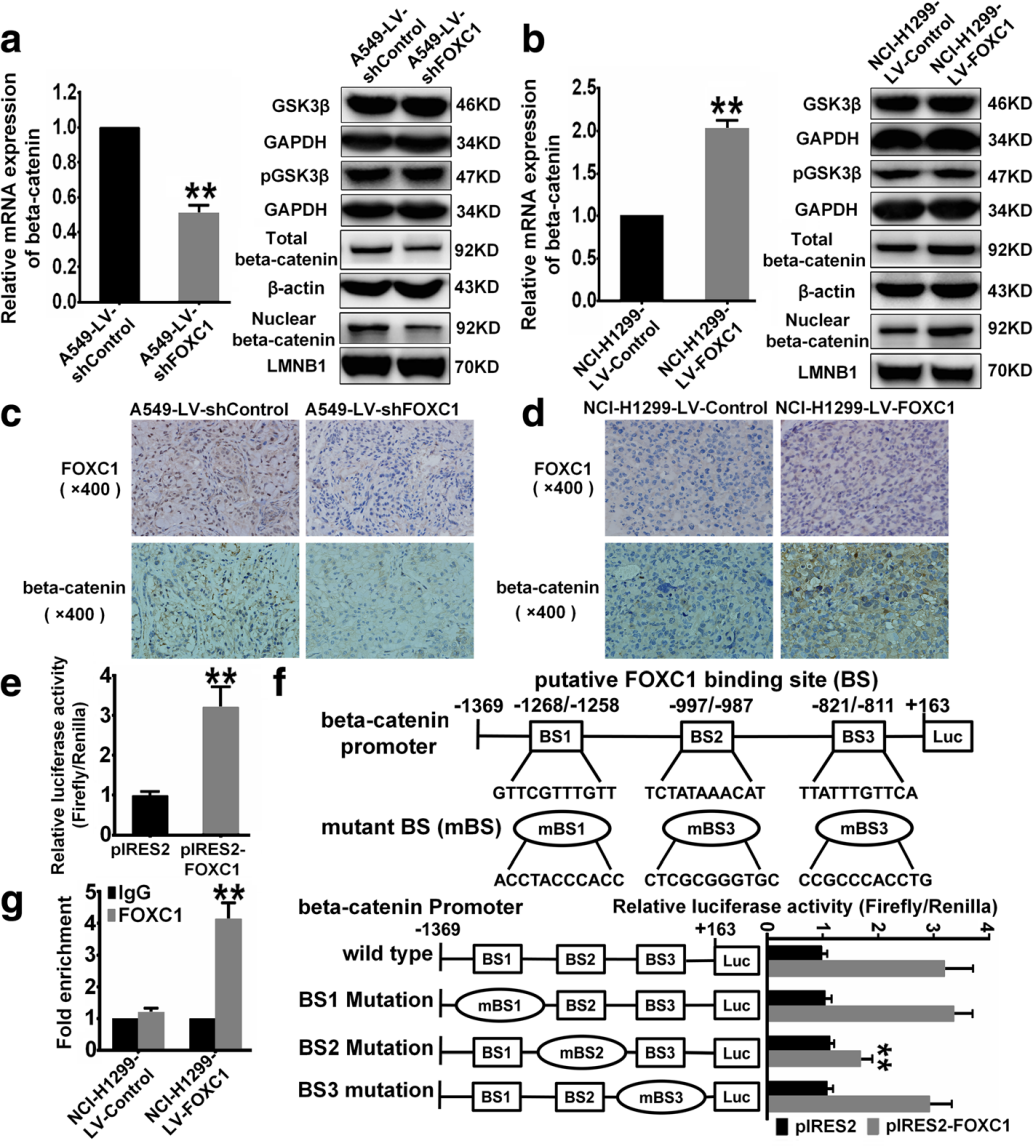
FOXC1 promotes beta-catenin expression by enhancing the activity of beta-catenin promoter. **a** and **b** Real-time PCR and western blotting were used to detect changes in beta-catenin mRNA, GSK3β, pGSK3β and total and nuclear beta-catenin protein levels induced by FOXC1 knockdown or overexpression. **c** and **d** Representative immunohistochemical staining of FOXC1 and beta-catenin in xenograft tumors. **e** Luciferase reporter assays showed that FOXC1 significantly enhanced beta-catenin promoter activity. **f** Putative FOXC1 binding sites in the beta-catenin promoter and mutations in corresponding binding sites. Schematic representations of wild-type and mutant beta-catenin promoters (left) and the corresponding relative luciferase activity (right) are shown. **g** ChIP and real-time PCR assays were used to investigate the binding of FOXC1 to the putative binding site BS2. All experiments were independently repeated three times. The bar graph presents the mean ± SD. **P < 0.01

Wnt/beta-catenin is an important signaling pathway for regulating CSCs [41]. Although alteration of FOXC1 expression did not cause significant changes in the levels of GSK3β and pGSK3β (Fig. 5a and b), FOXC1 knockdown reduced beta-catenin mRNA levels as well as levels of total and nuclear beta-catenin protein (Fig. 5a). FOXC1 knockdown also downregulated beta-catenin protein levels in xenograft tumors (Fig. 5c). FOXC1 overexpression increased beta-catenin mRNA levels and total and nuclear beta-catenin protein levels (Fig. 5b). Moreover, FOXC1 overexpression upregulated beta-catenin protein levels in xenograft tumors (Fig. 5d). Luciferase reporter assays showed that FOXC1 significantly enhanced beta-catenin promoter activity (Fig. 5e). Because of the conserved winged helix DNA-binding domain in FOX proteins [7], FOX transcription factor binding sites (BSs) share a consensus sequence: WAARYAAAYW (R = G or A, Y = C or T, W = T or A) [42, 43]. Analysis of the beta-catenin promoter sequence using TRANSFAC and JASPAR revealed three putative FOXC1 BSs: BS1, GTTCGTTTGTT [(− 1268/− 1258) beta-catenin, relative to the transcriptional start site]; BS2, TCTATAAACAT [(− 997/− 987) beta-catenin]; BS3, TTATTTGTTCA [(− 821/− 811) beta-catenin]. Although mutations in BS1 and BS3 did not significantly affect luciferase activity, BS2 mutation decreased FOXC1-induced luciferase activity (Fig. 5f). ChIP and real-time PCR assays confirmed the binding of FOXC1 to BS2 in NSCLC cells (Fig. 5g).

**Fig. 6:**
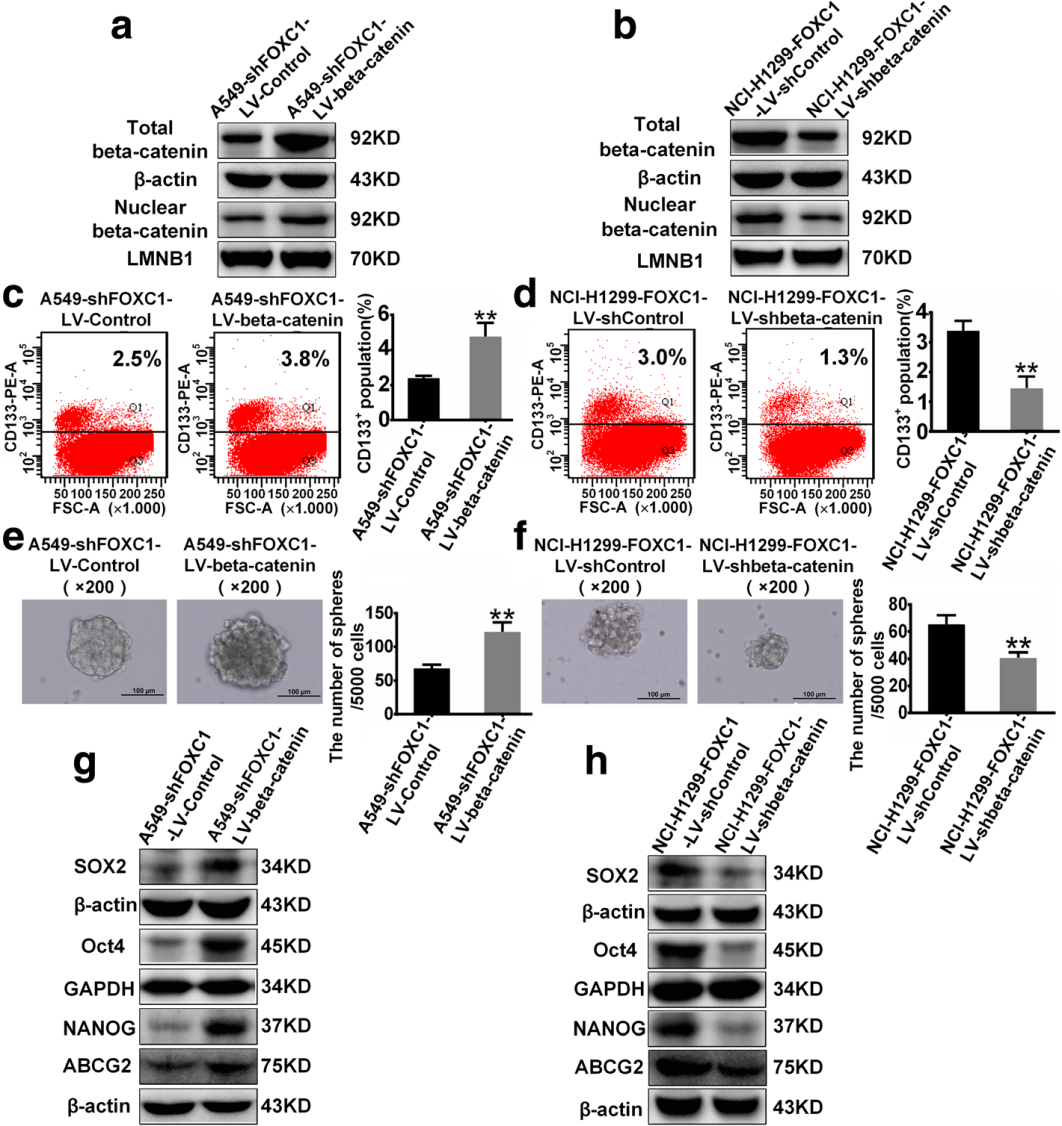
Beta-catenin mediates FOXC1-induced cancer stemness in NSCLC. **a** and **b** Total and nuclear beta-catenin protein levels were stably upregulated in A549-LV-shFOXC1 cells and downregulated in NCI-H1299-LV-FOXC1 cells. **c** and **d** The percentage of CD133^+^ cells was analyzed by flow cytometry. **e** and **f** Representative images (left) and numbers (right) of spheres (diameter > 100 μm). **g** and **h** Protein levels of SOX2, Oct4, NANOG and ABCG2. All experiments were independently repeated three times. The bar graph presents the mean ± SD. **P < 0.01

**Fig. 7:**
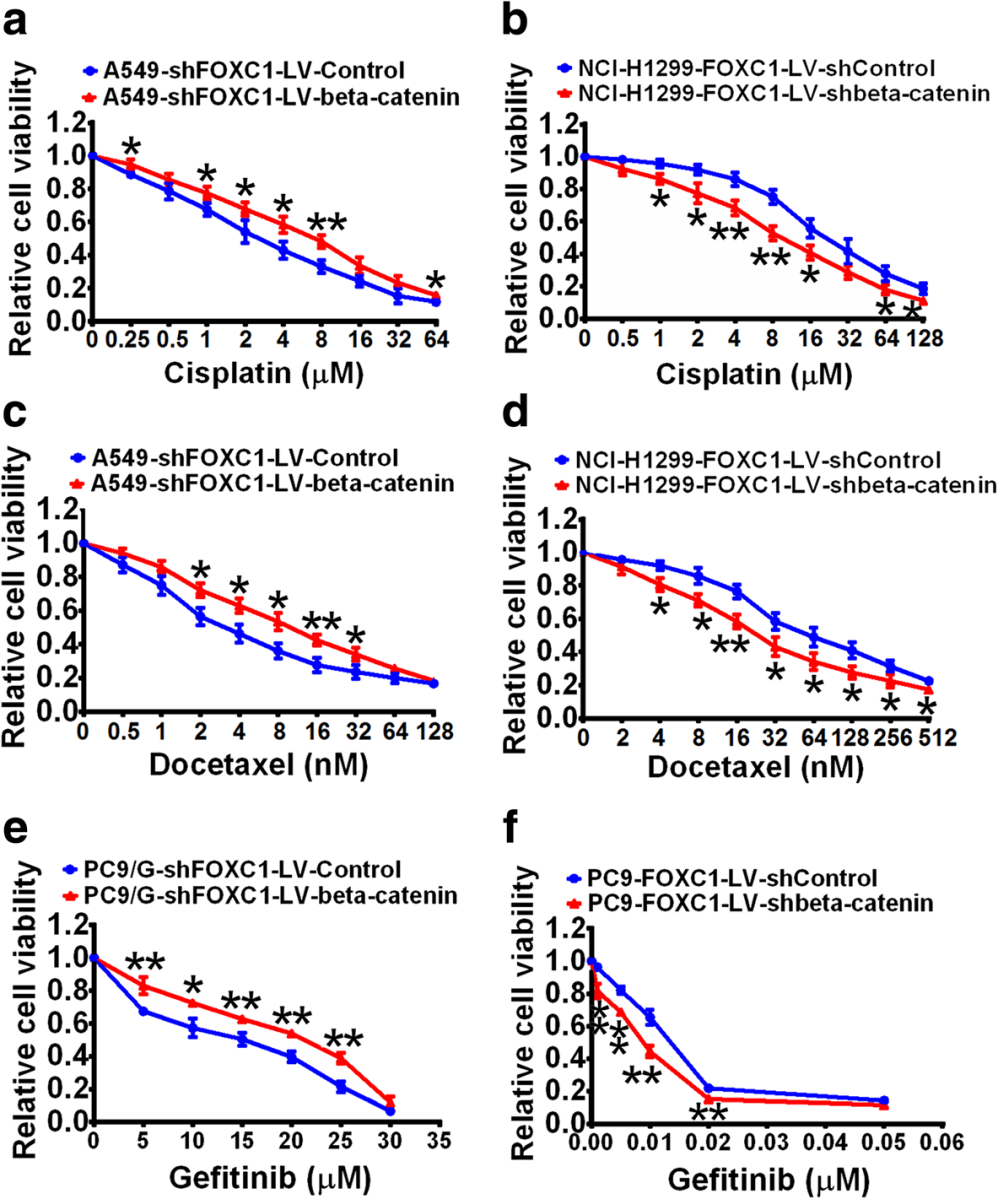
Beta-catenin mediates FOXC1-induced drug resistance in NSCLC. **a**, **c** and **e** Beta-catenin overexpression attenuated the inhibitory effects of cisplatin, docetaxel and gefitinib on the viability of FOXC1-knockdown cells. **b**, **d** and **f** Beta-catenin knockdown enhanced the cell killing effects of cisplatin, docetaxel and gefitinib on FOXC1-overexpressing cells. *P < 0.05, **P < 0.01

To investigate whether beta-catenin mediates FOXC1-induced CSC-like properties, LV-beta-catenin lentivirus was used to upregulate beta-catenin expression in A549-LV-shFOXC1 cells (Fig. 6a), and LV-shbeta-catenin lentivirus was used to knockdown beta-catenin expression in NCI-H1299-LV-FOXC1 cells (Fig. 6b). Beta-catenin overexpression increased CD133^+^ cell percentage (Fig. 6c), promoted sphere formation (Fig. 6e) and upregulated levels of SOX2, Oct4, NANOG and ABCG2 proteins (Fig. 6g) in FOXC1-knockdown cells. Conversely, beta-catenin knockdown decreased the percentage of CD133^+^ cells (Fig. 6d), inhibited sphere formation (Fig. 6f) and downregulated protein levels of SOX2, Oct4, NANOG and ABCG2 (Fig. 6h) in FOXC1-overexpressing cells. Moreover, beta-catenin overexpression attenuated the inhibitory effects of cisplatin, docetaxel and gefitinib on FOXC1-knockdown cell viability (Fig. 7a, c and e), whereas beta-catenin knockdown enhanced the inhibitory effects of drugs on the viability of FOXC1-overexpressing cells (Fig. 7b, d and f).

## Discussion

Elevated FOXC1 expression in NSCLC tissues and inverse correlation between FOXC1 expression and patient survival indicate that FOXC1 may be a negative prognostic factor in NSCLC. In this study, we found that FOXC1 is involved in the regulation of CSC-like properties, an important factor contributing to tumorigenesis and progression of NSCLC [6]. CD133 is a specific cell surface marker for CSCs in NSCLC, and CD133^+^ cells exhibit stem-like and highly tumorigenic features [44, 45]. Our results showed that FOXC1 knockdown decreased the percentage of CD133^+^ cells. Moreover, sphere-formation assay revealed that FOXC1 knockdown inhibited self-renewal ability, one of the most important characteristics of CSCs [4]. Oct4, NANOG, SOX2 and ABCG2 are essential stemness-related genes that maintain CSC-like properties in NSCLC [46–48], and FOXC1 knockdown decreased expression of these genes. Efficient tumorigenicity is a definitive feature of CSCs [49], and FOXC1 knockdown suppressed NSCLC cell tumorigenicity in vivo. Conversely, FOXC1 overexpression enhanced cancer stemness. These results indicate that FOXC1 is an important factor promoting cancer stemness in NSCLC.

Drug resistance, a major problem for NSCLC treatment [50, 51], is another essential property of CSCs, which resist therapy by enhancing membrane transporter activity and activating anti-apoptotic pathways. Conventional anti-cancer drugs kill most tumor cells but not CSCs, and surviving CSCs re-establish tumors [37]. Cisplatin and docetaxel are broadly employed cytotoxic anti-cancer agents in NSCLC [38, 39]. We found FOXC1 knockdown promoted NSCLC cell killing induced by cisplatin and docetaxel, whereas FOXC1 overexpression compromised this effect. These results indicate that FOXC1 plays a pivotal role in mediating the sensitivity of NSCLC cells to cisplatin and docetaxel and that it is a potential target for overcoming chemotherapy resistance in NSCLC. The molecularly targeted agent gefitinib, an epidermal growth factor receptor-tyrosine kinase inhibitor (EGFR-TKI), has been widely used in NSCLC treatment and it can dramatically improve patient survival. However, resistance to gefitinib severely reduces its clinical efficacy [52]. We detected higher levels of FOXC1 protein in gefitinib-resistant PC9/G cells than in gefitinib-sensitive PC9 cells. Knockdown of FOXC1 expression sensitized cells to gefitinib, whereas its overexpression conferred resistance to gefitinib. These results indicate that FOXC1 is a candidate biomarker and therapeutic target for overcoming EGFR-TKI resistance.

Finally, we explored the molecular mechanism underlying the promoting effects of FOXC1 on CSC-like properties. The Wnt/beta-catenin pathway is critical for regulating CSC-like properties. After activation of this pathway, increased amount of beta-catenin protein in the nucleus forms complexes with TCF/LEF to regulate target gene expression [41]. In NSCLC cells, FOXC1 knockdown reduced beta-catenin mRNA levels as well as total and nuclear beta-catenin protein levels. FOXC1 knockdown also downregulated beta-catenin protein levels in xenograft tumors. Moreover, FOXC1 knockdown decreased expression of Oct4, NANOG, SOX2 and ABCG2, which are important downstream target genes of beta-catenin in regulating cancer stemness [53–56]. In contrast, FOXC1 overexpression resulted in opposite effects. Luciferase reporter assays revealed that FOXC1 promoted beta-catenin expression by enhancing beta-catenin promoter activity. ChIP and real-time PCR assays confirmed the binding of FOXC1 to the beta-catenin promoter (− 997/− 987). Beta-catenin is considered to be a major contributor to high metastatic potential, a characteristic of CSCs [41, 57], and FOXC1 has been proven to promote metastasis in NSCLC [18, 19]. These observations are consistent with our finding that FOXC1 promotes beta-catenin expression. Furthermore, beta-catenin overexpression rescued the inhibited CSC-like properties induced by FOXC1 knockdown, whereas beta-catenin knockdown attenuated the enhanced CSC-like properties induced by FOXC1 overexpression. These results indicate that beta-catenin mediates the promoting effects of FOXC1 on CSC-like properties in NSCLC.

## Conclusions

In conclusion, this study is the first to demonstrate that FOXC1 contributes to CSC-like properties in NSCLC, including increased CD133^+^ cell population and stemness-related gene expression, enhanced self-renewal ability and tumorigenicity and induction of drug resistance, by promoting beta-catenin expression. These findings indicate that FOXC1 is a potential molecular target for anti-CSC-based therapies in NSCLC.

## Abbreviations

ABCG2: ATP-binding cassette subfamily G member 2
ChIP: Chromatin immunoprecipitation
CSC: Cancer stem cell
EGFR-TKI: Epidermal growth factor receptor-tyrosine kinase inhibitor
FOXC1: Forkhead box C1
GSK3β: Glycogen synthase kinase 3 beta
NSCLC: Non-small cell lung cancer
ShRNA: Short hairpin RNA
TCGA: The Cancer Genome Atlas
